# FDG PET/CT for Postoperative Surveillance in Malignant Pleural Mesothelioma: Temporal Evolution of Postsurgical Metabolic Activity and Diagnostic Performance for Recurrence Detection

**DOI:** 10.3390/cancers18122000

**Published:** 2026-06-19

**Authors:** Sun Ha Boo, Soo Jin Kwon, Seok Whan Moon, Yeon-Sil Kim, Sook-Hee Hong, Ie Ryung Yoo

**Affiliations:** 1 Division of Nuclear Medicine, Department of Radiology, Bucheon St. Mary’s Hospital, College of Medicine, The Catholic University of Korea, Seoul 06591, Republic of Korea; qneek0221@gmail.com; 2 Division of Nuclear Medicine, Department of Radiology, Eunpyeong St. Mary’s Hospital, College of Medicine, The Catholic University of Korea, Seoul 06591, Republic of Korea; 3 Department of Thoracic and Cardiovascular Surgery, Seoul St. Mary’s Hospital, College of Medicine, The Catholic University of Korea, Seoul 06591, Republic of Korea; 4 Department of Radiation Oncology, Seoul St. Mary’s Hospital, College of Medicine, The Catholic University of Korea, Seoul 06591, Republic of Korea; 5 Division of Medical Oncology, Department of Internal Medicine, Seoul St. Mary’s Hospital, College of Medicine, The Catholic University of Korea, Seoul 06591, Republic of Korea; 6 Division of Nuclear Medicine, Department of Radiology, Seoul St. Mary’s Hospital, College of Medicine, The Catholic University of Korea, Seoul 06591, Republic of Korea

**Keywords:** FDG PET/CT, malignant pleural mesothelioma, postoperative surveillance, recurrence, postsurgical change

## Abstract

**Background/Objectives:** Differentiating recurrent disease from postsurgical changes on ^18^F-fluorodeoxyglucose (FDG) positron emission tomography/computed tomography (PET/CT) remains challenging in malignant pleural mesothelioma (MPM). This study aimed to characterize the temporal patterns of postsurgical FDG uptake and evaluate the diagnostic performance of FDG PET/CT for detecting recurrent disease after radical surgery. **Methods:** We retrospectively analyzed 91 postsurgical PET/CT scans from 45 patients with MPM who underwent extrapleural pneumonectomy (EPP; *n* = 29) or pleurectomy/decortication (P/D; *n* = 16). Scans were stratified into four postoperative time intervals: <6 months, 6 to <12 months, 12 to <24 months, and ≥24 months. FDG uptake in the postsurgical bed and local recurrent lesions was quantified using maximum standardized uptake value ratios normalized to the mediastinal blood pool and liver. Recurrence was confirmed by histopathology or follow-up imaging. **Results:** Postsurgical FDG uptake showed a time-dependent decline, with significantly lower uptake beyond 24 months postoperatively (*p* < 0.05). EPP patients demonstrated significantly higher postsurgical FDG uptake than P/D patients (*p* < 0.01). FDG PET/CT identified occult recurrence in 23.4% of CT-negative scans. Local recurrent lesions showed significantly higher FDG uptake than postsurgical changes across all postoperative intervals (*p* < 0.001). **Conclusions**: Postsurgical FDG uptake in MPM demonstrates a time-dependent decline, and surgical extent is an important determinant of background metabolic activity. Despite this variable background, FDG PET/CT demonstrated high diagnostic accuracy for detecting recurrent disease, including CT-occult recurrences. Incorporating surgical type and postoperative interval into PET/CT interpretation may improve diagnostic accuracy in postoperative MPM surveillance.

## Simple Summary

Malignant pleural mesothelioma is an aggressive cancer of the pleural lining, often treated with radical surgery. After surgery, monitoring for cancer recurrence is essential but challenging, as postsurgical changes can mimic tumors on imaging. We examined how metabolic activity on positron emission tomography/computed tomography (PET/CT) evolves over time in the surgical site after radical surgery, and whether PET/CT can reliably distinguish recurrence from postsurgical changes. Metabolic activity in the surgical site gradually decreased over time, with significant reduction beyond two years after surgery. More extensive surgery was associated with higher background metabolic activity. Despite this variable background, recurrent tumors consistently showed higher metabolic activity than postsurgical changes. PET/CT also detected recurrence in nearly a quarter of cases appearing normal on conventional CT. These findings support the use of PET/CT for postoperative surveillance and highlight the importance of considering surgical type and time since surgery when interpreting scans.

## 1. Introduction

Malignant pleural mesothelioma (MPM) is the most common primary pleural malignancy, with a strong association with asbestos exposure [1]. Despite regulatory restrictions on asbestos use, the incidence of MPM continues to rise in many regions, owing to the prolonged latency period between exposure and disease onset [2]. The prognosis remains poor, with a median overall survival of <12 months following diagnosis and a 5-year overall survival rate of approximately 5% [3,4].

Multimodal treatment strategies incorporating surgery, chemotherapy, and radiotherapy represent the current standard of care for improving survival outcomes in selected patients with MPM [5,6]. Radical surgery includes either extrapleural pneumonectomy (EPP), involving en bloc resection of the lung, pleura, pericardium, and hemidiaphragm, or pleurectomy/decortication (P/D), a lung-sparing procedure aimed at maximal cytoreduction. These extensive procedures result in substantial anatomical and physiological alterations that complicate postoperative imaging interpretation and surveillance. Accurate postoperative surveillance is essential for the early detection of recurrence and timely treatment planning, yet remains a major clinical challenge in MPM.

Computed tomography (CT) is routinely used for postoperative surveillance; however, its ability to detect recurrence is substantially limited by the anatomical distortion, fibrosis, and pleural thickening that commonly follow radical resection [7,8,9]. ^18^F-fluorodeoxyglucose (FDG) positron emission tomography/computed tomography (PET/CT) offers complementary functional information that may improve recurrence detection beyond what CT alone can provide [10,11,12].

Nevertheless, FDG uptake in the surgical bed is not specific to malignancy and may reflect inflammatory and reparative processes associated with healing or prior radiotherapy [13,14]. In MPM, the extensive surgical trauma inherent to EPP or P/D may sustain and intensify this background metabolic activity [13], making it challenging to distinguish residual inflammation from early recurrence. Although several studies have evaluated the diagnostic performance of PET/CT in this setting [15,16,17,18], evidence regarding the temporal evolution of postsurgical FDG uptake and its impact on differentiating recurrence from postsurgical changes remains limited [15,17,18].

Therefore, this study aimed to characterize the time-dependent changes in FDG uptake within the surgical bed and to evaluate the diagnostic performance of FDG PET/CT for detecting recurrent disease in patients with MPM following radical surgery.

## 2. Materials and Methods

### 2.1. Patient Selection and Clinical Data Collection

This retrospective study was approved by the Institutional Review Board (IRB) of Seoul St. Mary’s Hospital (KC24RASI0346) and the requirement for informed consent was waived due to the retrospective nature of the study. Patients diagnosed with MPM who underwent radical surgery (EPP or P/D) at a single institution between March 2009 and December 2022 were identified. Patients were included if they had undergone one or more postoperative FDG PET/CT scans for clinical surveillance or evaluation of suspected recurrence. The institutional postoperative surveillance protocol comprised chest CT at 1–2 months after surgery, followed by subsequent imaging at 2–6-month intervals.

**Figure 1:**
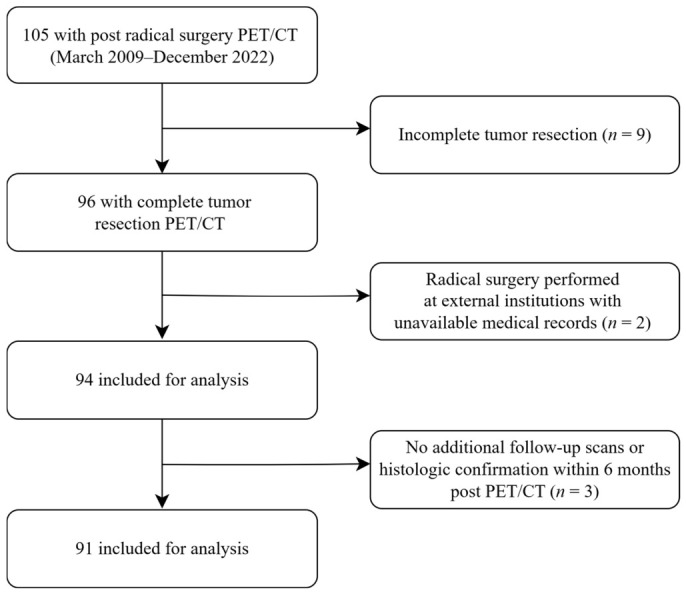
Flowchart of the study population. PET/CT, positron emission tomography/computed tomography.

Of the 105 FDG PET/CT scans initially identified, 14 were excluded for the following reasons: incomplete tumor resection (*n* = 9), surgery performed at external institutions with unavailable medical records (*n* = 2), or absence of follow-up imaging or histological confirmation within 6 months of the FDG PET/CT scan (*n* = 3). The remaining 91 FDG PET/CT scans from 45 patients were included in the diagnostic performance analysis (Figure 1). For the quantitative analysis of postsurgical FDG uptake, one scan was excluded because extensive diffuse recurrence throughout the surgical bed precluded reliable quantification of background metabolic activity; this scan was retained for the quantitative analysis of recurrent lesion FDG uptake. Accordingly, postsurgical bed activity was quantified in 90 scans from 44 patients, while recurrent lesion FDG uptake was quantified in 91 scans from 45 patients. Clinical data, including histopathological subtype, surgical approach, and neoadjuvant or adjuvant therapy history, were retrieved from medical records.

The treatment strategies were individualized, with surgery combined with chemotherapy, radiotherapy, or both according to each patient’s clinical profile. Chemotherapy consisting of cisplatin and pemetrexed was administered as neoadjuvant or adjuvant treatment. Radiotherapy was delivered preoperatively (20–30 Gy) or postoperatively (39–63 Gy).

### 2.2. FDG PET/CT Imaging Protocol

All patients fasted for at least 6 h prior to the FDG PET/CT examination, and blood glucose levels were measured and confirmed to be below 200 mg/dL before radiotracer administration. PET/CT imaging was performed 60 min after the intravenous administration of FDG at a dose of 3.7–5.5 MBq/kg. Scans were acquired using one of the following two PET/CT systems: Biograph TruePoint (Siemens Medical Solutions, Erlangen, Germany), or Discovery 710 (GE Healthcare, Chicago, IL, USA). CT acquisition without contrast was performed from the skull vertex to the proximal thigh using the following parameters: 120 kVp, 50 mAs, and 5 mm slice thickness (Biograph TruePoint); 120 kVp, auto-mAs modulated by tomogram, and 2.5 mm slice thickness (Discovery 710). PET images were subsequently acquired over the same anatomical coverage. PET images were reconstructed using a standard ordered-subset expectation-maximization algorithm with CT-based attenuation correction.

### 2.3. Image Analysis

Two board-certified nuclear medicine physicians, with 7 and 20 years of experience in PET/CT interpretation, independently reviewed all FDG PET/CT images using Mirada XD3 software (Mirada Medical, Oxford, UK). Both readers were blinded to clinical, histopathological, and follow-up information at the time of image review. Discrepancies were resolved by consensus.

For analysis, the FDG PET/CT scans were stratified into four postsurgical time periods: less than 6 months (P1), 6 to less than 12 months (P2), 12 to less than 24 months (P3), and 24 months or more (P4).

Local recurrence was defined as focal FDG uptake exceeding the surrounding background activity of the postsurgical bed. Regional nodal metastasis was defined as focal FDG uptake exceeding mediastinal blood pool activity within a lymph node, regardless of its size on CT. Distant metastasis was defined as focal FDG uptake at sites outside the ipsilateral hemithorax inconsistent with physiological tracer distribution. Focal FDG-positive lesions at all sites were classified as suspicious for recurrence regardless of corresponding CT findings. Lesion classification was based on visual assessment using the site-specific FDG uptake criteria described above, without applying a fixed numerical SUV cutoff. Diffuse or linear FDG uptake without nodular CT abnormalities was interpreted as benign postsurgical changes.

Semi-quantitative FDG uptake analysis was performed for local recurrence and postsurgical changes within the surgical bed. The maximum standardized uptake value (SUVmax) was measured by placing a three-dimensional volume of interest (VOI) over the area of highest FDG uptake within the postsurgical bed and each suspected local recurrent lesion. Normalized SUVmax ratios were calculated using mediastinal blood pool (BP) and liver (L) as reference regions. BP activity was measured as the mean standardized uptake value (SUVmean) of three 1-cm^3^ spherical VOIs placed within the aortic arch, and L activity as the SUVmean of three 3-cm^3^ spherical VOIs drawn within the right hepatic lobe. Four normalized FDG uptake ratios were calculated: postsurgical bed SUVmax to BP SUVmean (OP/BP ratio), postsurgical bed SUVmax to L SUVmean (OP/L ratio), local recurrent lesion SUVmax to BP SUVmean (lesion/BP ratio), and local recurrent lesion SUVmax to L SUVmean (lesion/L ratio).

Histopathological confirmation served as the definitive reference standard when available (*n* = 10). In cases where tissue sampling was not feasible, recurrence was confirmed by unequivocal radiological progression, defined as new lesions or progressive enlargement of suspected findings, on follow-up CT, magnetic resonance imaging (MRI), or FDG PET/CT (*n* = 41). Lesions were classified as benign if subsequent imaging demonstrated stability or regression of suspected findings.

Recurrent lesions detected on FDG PET/CT were classified by anatomical distribution as local recurrence (ipsilateral hemithorax), regional nodal recurrence, or distant metastasis (intrathoracic or extrathoracic).

### 2.4. Statistical Analysis

Normality of continuous variables was assessed using the Shapiro–Wilk test. As PET parameters did not conform to a normal distribution, non-parametric tests were applied. Data are presented as mean ± standard deviation. All statistical analyses were performed using IBM SPSS software (version 30.0; IBM Corp., Armonk, NY, USA).

The Kruskal–Wallis test was used to compare PET parameters across the four postoperative time intervals. The Mann–Whitney U tests were additionally performed to compare PET parameters between earlier (P1–P3) and later (P4) postoperative periods, to compare postsurgical metabolic activity between surgical types (EPP vs. P/D) and between radiotherapy groups (irradiated vs. non-irradiated), and to compare FDG uptake between recurrent lesions and postsurgical changes.

The diagnostic performance of FDG PET/CT for detecting recurrence, including local recurrence, regional nodal metastasis, and distant metastasis, was evaluated using sensitivity, specificity, positive predictive value (PPV), negative predictive value (NPV), and accuracy at the scan level (*n* = 91) with 95% confidence intervals. A patient-level analysis restricted to the first postoperative PET/CT scan per patient (*n* = 45) was additionally performed to account for the potential non-independence arising from multiple scans per patient. Statistical significance was defined as *p* < 0.05.

## 3. Results

### 3.1. Patient Characteristics

A total of 45 patients (30 male and 15 female) were included, contributing 91 postoperative FDG PET/CT scans for analysis. The median age was 65 years (range, 35–76 years). Of the 45 patients, 29 (64.4%) underwent EPP and 16 (35.6%) underwent P/D. The histopathological subtypes included epithelioid (*n* = 29), biphasic (*n* = 13), sarcomatoid (*n* = 2), and desmoplastic (*n* = 1).

The number of FDG PET/CT scans per patient varied as follows: one scan in 20 patients, two scans in 10, three scans in 10, four scans in four, and five scans in one patient. Radiotherapy was administered to 62.2% (28/45) of patients, with a higher proportion in the EPP group (82.8%, 24/29) than in the P/D group (25.0%, 4/16). Patient characteristics are summarized in Table 1.

### 3.2. Postsurgical FDG Uptake: Temporal Trends

The 91 FDG PET/CT scans were distributed across four postoperative time intervals: P1 (*n* = 19), P2 (*n* = 21), P3 (*n* = 29), and P4 (*n* = 21).

**Figure 2:**
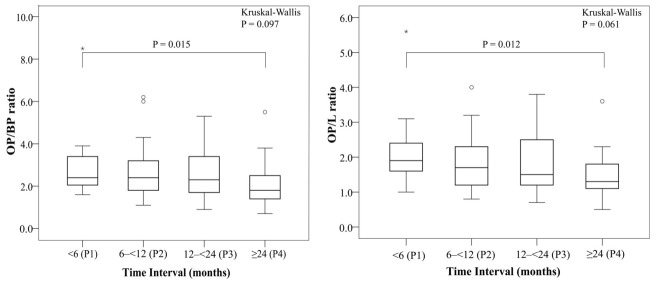
Postsurgical bed activity across different time intervals. Overall comparisons among the four postoperative intervals (P1–P4) were performed using the Kruskal–Wallis test. The bracket indicates the Mann–Whitney U test comparing the P4 (≥24 months) with the combined earlier postoperative intervals (P1–P3). The corresponding *p*-value is shown above the bracket. Circles (○) indicate mild outlier values; asterisks (*) indicate extreme outlier values. OP/BP ratio, postsurgical bed activity SUVmax to blood pool SUVmean; OP/L ratio, postsurgical bed activity SUVmax to liver SUVmean; SUVmax, maximum standardized uptake value; SUVmean, mean standardized uptake value.

**Figure 3:**
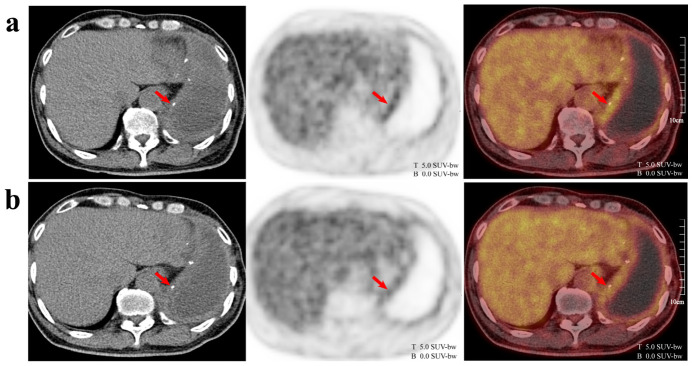
Time dependent reduction in postsurgical FDG uptake. A 64-year-old man underwent extrapleural pneumonectomy for malignant pleural mesothelioma. ^18^F-fluorodeoxyglucose (FDG) positron emission tomography/computed tomography (PET/CT) scans at 12 months (**a**) and 22 months (**b**) postoperatively showed a progressive decrease in FDG uptake within the surgical bed over time (arrows). Spatial scale bars (10 cm) and FDG uptake intensity scales (0.0–5.0 SUV-bw) are displayed for reference. T and B indicate the top (maximum, 5.0 SUV-bw) and bottom (minimum, 0.0 SUV-bw) of the FDG uptake intensity scale, respectively.

In the scan-based analysis (*n* = 90), the OP/BP ratio was 2.9 ± 1.5 at P1, 2.7 ± 1.4 at P2, 2.7 ± 1.2 at P3, and 2.1 ± 1.1 at P4. A similar decreasing trend was observed in the OP/L ratio: 2.1 ± 1.0 at P1, 1.9 ± 0.8 at P2, 1.8 ± 0.8 at P3, and 1.4 ± 0.7 at P4 (Figure 2 and Figure 3).

Although the overall comparison across the four time intervals did not reach statistical significance (*p* = 0.097 for OP/BP; *p* = 0.061 for OP/L), the comparison between P4 (≥24 months) and the combined earlier intervals (P1–P3) revealed statistically significant reduction in both OP/BP (2.8 ± 1.4 vs. 2.1 ± 1.1; *p* = 0.015) and OP/L (1.9 ± 0.9 vs. 1.4 ± 0.7; *p* = 0.012) ratios (Table 2). At the patient-level analysis (*n* = 44), a consistent downward trend was similarly observed; however, statistical significance was not reached, partly owing to the smaller sample size in the later time periods (Supplementary Table S1).

The distribution of histological subtypes, surgical type, and radiotherapy status appeared broadly similar across all four time intervals (Supplementary Table S2).

### 3.3. Impact of Surgical Type and Radiotherapy

A total of 63 FDG PET/CT scans were performed after EPP and 27 after P/D. Postsurgical metabolic activity was significantly higher in the EPP group than in the P/D group for both the OP/BP ratio (2.9 ± 1.4 vs. 2.0 ± 0.8; *p* = 0.002) and OP/L ratio (2.0 ± 0.9 vs. 1.4 ± 0.6; *p* = 0.001) (Table 2).

Of the 90 scans, 53 were performed after radiotherapy and 37 without prior radiotherapy. The post-radiotherapy group demonstrated significantly higher postsurgical FDG uptake than the non-radiotherapy group, with higher OP/BP (3.0 ± 1.5 vs. 2.0 ± 0.8; *p* < 0.001) and OP/L (2.1 ± 0.9 vs. 1.4 ± 0.6; *p* < 0.001) ratios (Table 2). The interval between radiotherapy completion and FDG PET/CT acquisition was variable, with a median of 12.1 months (range, 2.3–46.5 months).

To further delineate the independent contributions of surgical type and radiotherapy, stratified analyses were performed. Within the EPP group, postsurgical FDG uptake was higher in patients who received radiotherapy than in those who did not; however, this difference reached significance only for the OP/L ratio (*p* = 0.022) and not for the OP/BP ratio (*p* = 0.099) (Table 2). Among patients who did not receive radiotherapy, those who underwent EPP showed significantly higher postsurgical FDG uptake than those who underwent P/D for the OP/BP ratio (*p* = 0.012), although this difference did not reach significance for the OP/L ratio (*p* = 0.089) (Table 2).

### 3.4. Recurrence Detection and Diagnostic Performance

Of the 91 PET/CT scans, disease recurrence was confirmed in 49 scans (53.8%), including local recurrence in 44 (89.8%). The remaining 42 scans (46.2%) were classified as benign postsurgical changes. The characteristics and anatomical distribution of the recurrent lesions are summarized in Supplementary Tables S3 and S4.

Among the 91 scans, 44 (48.4%) showed CT findings suspicious for recurrence, whereas the remaining 47 (51.6%) had no CT findings suspicious for recurrence. Among the 44 CT-positive scans, FDG PET/CT confirmed recurrence in 38 (86.4%) and reclassified the remaining 6 (13.6%) as benign postsurgical changes. In contrast, among the 47 CT-negative scans, FDG PET/CT identified focal FDG uptake suspicious for recurrent disease in 13 cases. Of these, 11 were confirmed as true recurrences on follow-up, including local recurrence, regional nodal involvement, and distant metastases not identified on CT. Among these 11 CT-occult recurrences, 4 also showed concurrent nodal or distant metastases beyond local recurrence. The remaining 2 cases were subsequently confirmed as false positives on follow-up: one represented reactive lymphoid hyperplasia resembling a supraclavicular nodal metastasis at 5 months postoperatively, and the other represented inflammatory changes in the pericardial area mimicking local recurrence at 13 months postoperatively.

Table 3 summarizes the relationship between CT findings, FDG PET/CT findings, and the final recurrence status. For this analysis, recurrence was defined as any recurrent disease, including local recurrence, regional nodal metastasis, and distant metastasis. The sensitivity, specificity, PPV, NPV, and accuracy for detecting recurrence were 100.0%, 95.2%, 96.1%, 100.0%, and 97.8%, respectively. A sensitivity analysis according to the method of recurrence confirmation is presented in Supplementary Table S5. Among the 11 examinations with histopathological confirmation, FDG PET/CT demonstrated a sensitivity of 100.0%, specificity of 33.3%, and accuracy of 81.8%. Among the 80 examinations confirmed by serial imaging follow-up, FDG PET/CT demonstrated a sensitivity, specificity, and accuracy of 100.0%.

At the patient level, when the analysis was restricted to the first postoperative scan per patient, diagnostic performance remained robust and consistent with the scan-level results, with a sensitivity of 100.0% (19/19) and specificity of 96.2% (25/26). Detailed patient-level metrics are provided in Supplementary Table S6.

### 3.5. Quantitative Comparison: Postsurgical Changes vs. Local Recurrence

**Figure 4:**
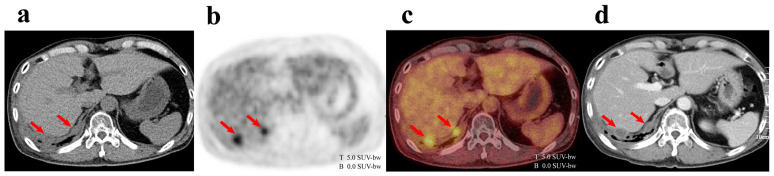
Local recurrence detected on ^18^F-fluorodeoxyglucose (FDG) positron emission tomography/computed tomography (PET/CT). A 49-year-old man underwent pleurectomy/decortication for malignant pleural mesothelioma. FDG PET/CT performed at 10 months postoperatively (**a**–**c**) demonstrated focal FDG uptake in the postsurgical bed of the right lower hemithorax, with focal pleural thickening on CT (arrows), consistent with local recurrence. Follow-up chest CT obtained 3 months later confirmed progressive nodular pleural thickening (**d**). Spatial scale bars (10 cm) and FDG uptake intensity scales (0.0–5.0 SUV-bw) are displayed for reference. T and B indicate the top (maximum, 5.0 SUV-bw) and bottom (minimum, 0.0 SUV-bw) of the FDG uptake intensity scale, respectively.

In the scan-based analysis, the OP/BP and OP/L ratios for postsurgical changes were 2.6 ± 1.3 and 1.8 ± 0.9, respectively. In contrast, the lesion/BP and lesion/L ratios for recurrent lesions were significantly higher, at 4.3 ± 1.8 and 3.0 ± 1.3, respectively (both *p* < 0.001; Figure 4; Table 4). This significant difference was consistently observed across both the EPP and P/D subgroups (*p* < 0.001 for all comparisons; Table 4). At the patient level, restricting the analysis to the first scan per patient (*n* = 45), recurrent lesions similarly demonstrated significantly higher FDG uptake than postsurgical changes across all ratio metrics (*p* < 0.001; Supplementary Table S7).

## 4. Discussion

In this study, we characterized the temporal evolution of postsurgical metabolic activity in patients with MPM following radical surgery and evaluated the diagnostic performance of FDG PET/CT for detecting recurrent disease. Postsurgical FDG uptake demonstrated a time-dependent decline, with a significant reduction beyond 24 months postoperatively. EPP was associated with significantly higher background metabolic activity than P/D. Despite this elevated and temporally variable background, recurrent lesions consistently exhibited significantly higher FDG uptake than benign postsurgical changes, supporting the diagnostic utility of FDG PET/CT as a postoperative surveillance tool. Furthermore, FDG PET/CT demonstrated high diagnostic accuracy for detecting recurrent disease and provided additional clinical value by clarifying indeterminate CT findings and identifying occult recurrences not visualized on CT.

FDG uptake is not specific to malignancy and can reflect a wide range of inflammatory and reparative processes [19], necessitating careful interpretation in the postoperative setting. Persistent postsurgical FDG uptake is primarily driven by inflammatory processes, including macrophage activation and fibroblast proliferation during tissue healing [20]. In patients undergoing extensive procedures such as EPP or P/D, this elevated metabolic activity may reflect persistent inflammation and fibrotic remodeling along the resected tissue planes [13]. In the present study, measurable postsurgical FDG uptake was observed across all time intervals, including in scans acquired beyond 24 months after surgery. This suggests that low-level background activity can persist well after surgery, with direct relevance to FDG PET/CT interpretation in postoperative surveillance. Nevertheless, this metabolic activity diminishes progressively over time, and the gradual improvement in lesion-to-background contrast may facilitate more confident PET/CT interpretation during follow-up. Notably, the majority of local recurrences in our cohort were detected during the early postoperative period, when postsurgical background activity remained elevated. Despite this, PET/CT remained reliable for recurrence detection even under challenging conditions. Of note, the distribution of histological subtypes appeared broadly similar across all postoperative time intervals, with epithelioid subtype comprising 65–67% of scans in each group (Supplementary Table S2). This observation suggests that the lower postsurgical FDG uptake observed in the late postoperative period (P4) is unlikely to be explained solely by enrichment of patients with favorable tumor biology. Nevertheless, residual survivor-selection bias cannot be excluded given the retrospective nature of the study.

Beyond temporal changes, the extent of surgical resection emerged as an important determinant of postsurgical FDG uptake. Postsurgical metabolic activity was significantly higher in EPP patients than in P/D patients, likely reflecting the more extensive surgical trauma, tissue disruption, and mediastinal manipulation associated with EPP. As a more radical procedure, EPP elicits a more robust and prolonged inflammatory response compared with P/D, a lung-sparing procedure associated with less extensive tissue disruption. The contribution of radiotherapy to postsurgical FDG uptake appeared more variable. Radiotherapy can induce inflammatory changes and fibrosis that persist for 6–12 months, and sometimes up to 2 years [14,21,22], potentially contributing to elevated metabolic activity in the surgical bed. Variability in the interval between radiotherapy completion and FDG PET/CT acquisition may have further contributed to the heterogeneity in postsurgical uptake observed among irradiated patients. In our stratified analysis, even among patients who did not receive radiotherapy, EPP was associated with significantly higher postsurgical uptake compared with P/D for the OP/BP ratio (*p* = 0.012), although this difference did not reach significance for the OP/L ratio (*p* = 0.089). These findings suggest that surgical trauma may contribute independently to elevated background metabolic activity. Given that both surgical trauma and radiation-induced changes can mimic malignant FDG uptake, surgical type and radiotherapy history should be carefully considered during postoperative FDG PET/CT interpretation. This consideration may be particularly important in EPP patients, in whom higher background metabolic activity is expected.

Few studies have evaluated postsurgical FDG uptake in MPM using normalized metabolic ratios rather than absolute SUVmax. While SUVmax is widely used for quantitative PET/CT analysis [23,24], absolute SUV measurements can be influenced by multiple factors, including patient weight, blood glucose level, scan timing, and inter-scanner differences [25], potentially compromising reproducibility in longitudinal studies. This limitation is particularly relevant in the present study, which spanned multiple years and included scans acquired using two different PET/CT systems. Reliance on absolute SUVmax alone could therefore have introduced systematic measurement variability that obscured true longitudinal differences in postsurgical FDG uptake. By normalizing FDG uptake to stable reference regions, such as the mediastinal blood pool and liver, this approach provided more reproducible quantitative metrics [26], enabling reliable longitudinal comparison between postsurgical inflammatory changes and recurrent lesions. Normalized metabolic ratios were significantly higher in scans with local recurrence than in those with postsurgical changes across all subgroups, including both EPP and P/D patients, consistent with and extending findings from a prior study using absolute SUVmax [17]. However, a degree of overlap in quantitative values was observed between the two groups, underscoring that fixed cutoffs should be regarded as supportive rather than definitive diagnostic criteria, and that quantitative PET parameters must be interpreted alongside morphological and clinical context.

CT is commonly used for postoperative surveillance in MPM patients; however, its ability to detect recurrence is limited by the anatomical distortion caused by surgery, subsequent fibrosis, and pleural thickening, all of which can obscure or mimic the appearance of recurrence [27]. In the present study, FDG PET/CT identified occult recurrence in 11 of 47 CT-negative scans (23.4%), including local recurrences, regional nodal involvement, and distant metastases not visualized on CT. Conversely, PET/CT reclassified six CT-positive scans as benign postsurgical changes, potentially avoiding unnecessary intervention. These results are consistent with prior studies demonstrating that PET/CT improves recurrence detection compared with CT alone in the postoperative setting [15,17,18], and further extend those findings by quantifying the frequency and clinical relevance of CT-occult recurrences. Beyond local recurrence detection, distant metastatic disease was identified in 23 of 49 recurrence-confirmed scans (46.9%), representing nearly half of all confirmed recurrences. This finding has important implications for treatment planning, as the presence of distant metastases may preclude further local therapy and necessitate systemic treatment. It also underscores the advantage of FDG PET/CT as a whole-body imaging modality capable of providing a comprehensive assessment of disease burden. Nevertheless, FDG PET/CT interpretation in the postoperative setting remains challenging. Granulation tissue along resection margins can exhibit irregular, nodular FDG uptake that overlaps with malignant uptake patterns [28], and some recurrent tumors may demonstrate relatively low metabolic activity owing to small lesion size, histological subtype, prior treatment effects, or necrosis [29,30]. Although focal FDG uptake without corresponding CT abnormalities should not be dismissed in the postoperative MPM setting, FDG PET/CT findings should be interpreted in conjunction with morphological CT findings, clinical history, and postoperative timeline to minimize false-positive interpretation and unnecessary intervention.

Several limitations of this study warrant consideration. First, the retrospective, single-center design and relatively small sample size may limit the generalizability of the findings to broader patient populations. Second, the inclusion of multiple PET/CT scans per patient may introduce statistical non-independence. Although a patient-level subgroup analysis restricted to the first postoperative scan per patient yielded consistent results, this limitation should be considered when interpreting the primary findings. Third, the relatively small number of scans acquired during the late postoperative period (≥24 months) may have limited the statistical power of the temporal analysis. Fourth, histopathological confirmation was not obtained in all cases; where tissue sampling was not feasible, recurrence was defined by unequivocal radiological progression on follow-up imaging. Although this approach reflects real-world clinical practice, it may introduce verification bias. Furthermore, because follow-up imaging included PET/CT in some cases, incorporation bias cannot be fully excluded. Consistent with this possibility, specificity and accuracy were substantially lower in the histopathologically confirmed subgroup (33.3% and 81.8%, respectively) than in the imaging-confirmed subgroup (both 100.0%), although sensitivity remained 100.0% in both subgroups. Therefore, the overall diagnostic accuracy reported in this study, which is largely driven by the imaging-confirmed subgroup, should be interpreted in light of this potential incorporation bias. Fifth, quantitative PET analysis may be less reliable in patients with diffuse or miliary patterns of recurrence, where recurrent tumor uptake can overlap with postoperative inflammatory activity. In such cases, delineation of representative lesion and background regions may be challenging. Indeed, one examination in our cohort was excluded from postsurgical bed activity analysis because extensive diffuse recurrence throughout the surgical bed precluded reliable quantification. Therefore, visual interpretation in conjunction with CT findings remains important when evaluating non-focal recurrence patterns. Finally, heterogeneity in treatment protocols, particularly in the type and timing of radiotherapy, as well as variability in the interval between treatment completion and FDG PET/CT acquisition, may have influenced the extent of postsurgical FDG uptake. Although subgroup analyses were conducted to partially address this, residual confounding cannot be excluded.

Prospective multicenter studies with larger, more homogeneous patient cohorts are needed to validate the temporal patterns of postsurgical FDG uptake and the independent effect of surgical extent on background metabolic activity identified in this study. Future work should also investigate whether incorporating surgical type and postoperative interval into FDG PET/CT interpretation improves diagnostic accuracy and facilitates treatment decision-making during postoperative MPM surveillance.

## 5. Conclusions

Postsurgical FDG uptake in MPM demonstrates a time-dependent decline, and the extent of surgical resection is an important determinant of background metabolic activity. Despite this temporally variable and procedure-dependent background, FDG PET/CT demonstrated high diagnostic accuracy for detecting recurrent disease, including occult recurrences not visualized on CT. These findings support the clinical utility of FDG PET/CT for postoperative surveillance in MPM and highlight the importance of incorporating surgical type and postoperative interval into FDG PET/CT interpretation to optimize diagnostic accuracy.

## Figures and Tables

**Table 1:** Patient characteristics.

Characteristic		N (*n* = 45)	%
**Age (years)**			
Median (range)		65 (35–76)	
**Sex**			
Male		30	66.7
Female		15	33.3
**Histological subtype**			
Epithelioid		29	64.4
Biphasic		13	28.9
Sarcomatoid		2	4.4
Desmoplastic		1	2.2
**Previous therapy**			
	EPP (*n* = 29)	P/D (*n* = 16)	Total (*n* = 45)
Surgery alone	4 (13.8)	1 (6.3)	5 (11.1)
neo-CTx	25 (86.2)	16 (100.0)	41 (91.1)
neo-RTx	21 (72.4)	3 (18.8)	24 (53.3)
adj-CTx	6 (20.7)	7 (43.8)	13 (28.9)
adj-RTx	6 (20.7)	1 (6.3)	7 (15.6)

Values are presented as *n* (%). Percentages may not sum to 100% as individual patients may have received multiple treatment modalities. EPP, extrapleural pneumonectomy; P/D, pleurectomy/decortication; neo, neoadjuvant; adj, adjuvant; CTx, chemotherapy; RTx, radiotherapy.

**Table 2:** PET parameters by time interval and surgical type.

		PET Parameter (Range)	
		OP/BP Ratio	OP/L Ratio
**Time interval**	P1 (<6 months)	2.9 ± 1.5 (1.6–8.5)	2.1 ± 1.0 (1.0–5.6)
	P2 (6–<12 months)	2.7 ± 1.4 (1.1–6.2)	1.9 ± 0.8 (0.8–4.0)
	P3 (12–<24 months)	2.7 ± 1.2 (0.9–5.3)	1.8 ± 0.8 (0.7–3.8)
	P4 (≥24 months)	2.1 ± 1.1 (0.7–5.5)	1.4 ± 0.7 (0.5–3.6)
	*p*-value	0.097	0.061
**Time interval**	P1–P3 combined (<24 months)	2.8 ± 1.4 (0.9–8.5)	1.9 ± 0.9 (0.7–5.6)
	P4 (≥24 months)	2.1 ± 1.1 (0.7–5.5)	1.4 ± 0.7 (0.5–3.6)
	*p*-value	0.015	0.012
**Surgical type**	EPP	2.9 ± 1.4 (1.0–8.5)	2.0 ± 0.9 (0.6–5.6)
	P/D	2.0 ± 0.8 (0.7–3.7)	1.4 ± 0.6 (0.5–2.7)
	*p*-value	0.002	0.001
**Radiotherapy**	Post-radiotherapy	3.0 ± 1.5 (1.0–8.5)	2.1 ± 0.9 (0.8–5.6)
	Non-radiotherapy	2.0 ± 0.8 (0.7–4.0)	1.4 ± 0.6 (0.5–2.7)
	*p*-value	<0.001	<0.001
**Within EPP**	Post-radiotherapy	3.1 ± 1.5 (1.0–8.5)	2.1 ± 0.9 (0.8–5.6)
	Non-radiotherapy	2.3 ± 0.9 (1.1–4.0)	1.6 ± 0.7 (0.6–2.7)
	*p*-value	0.099	0.022
**Within non-radiotherapy**	EPP	2.3 ± 0.8 (1.1–4.0)	1.6 ± 0.7 (0.6–2.7)
	P/D	1.7 ± 0.7 (0.7–3.2)	1.2 ± 0.5 (0.5–2.3)
	*p*-value	0.012	0.089

PET, positron emission tomography; OP/BP ratio, postsurgical bed activity SUVmax to blood pool SUVmean; OP/L ratio, postsurgical bed activity SUVmax to liver SUVmean; EPP, extrapleural pneumonectomy; P/D, pleurectomy/decortication.

**Table 3:** Distribution of CT and FDG PET/CT findings according to recurrence status (*n* = 91).

CT	PET	Recurrence Status	N (%)
Positive	Positive	Recurrence	38 (41.8)
Negative	Positive	Recurrence	11 (12.1)
Negative	Positive	No recurrence	2 (2.2)
Positive	Negative	No recurrence	6 (6.6)
Negative	Negative	No recurrence	34 (37.4)

CT, computed tomography; PET, positron emission tomography; FDG, ^18^F-fluorodeoxyglucose.

**Table 4:** PET parameters of postsurgical bed and recurrent lesions.

		PET Parameters (Range)	
		Blood Pool (BP) Ratio	Liver (L) Ratio
**All PET/CT**	Postsurgical bed	2.6 ± 1.3 (0.7–8.5)	1.8 ± 0.9 (0.5–5.6)
	Recurrent lesion	4.3 ± 1.8 (1.4–9.0)	3.0 ± 1.3 (0.8–5.9)
	*p*-value	<0.001	<0.001
**EPP group**	Postsurgical bed	2.9 ± 1.4 (1.0–8.5)	2.0 ± 0.9 (0.6–5.6)
	Recurrent lesion	4.4 ± 1.9 (1.4–9.0)	3.0 ± 1.3 (0.8–5.9)
	*p*-value	<0.001	<0.001
**P/D group**	Postsurgical bed	2.0 ± 0.8 (0.7–3.7)	1.4 ± 0.6 (0.5–2.7)
	Recurrent lesion	3.4 ± 1.6 (2.0–7.6)	2.9 ± 1.1 (1.6–5.2)
	*p*-value	<0.001	<0.001

PET, positron emission tomography; EPP, extrapleural pneumonectomy; P/D, pleurectomy/decortication.

## Data Availability

The data used in this study are not publicly available due to patient privacy concerns and institutional restrictions but are available from the corresponding author upon reasonable request.

## Supplementary Materials

The following supporting information can be downloaded at https://www.mdpi.com/article/10.3390/cancers18122000/s1, Table S1: Postsurgical FDG uptake by time interval (patient-level analysis); Table S2: Distribution of histological subtypes, surgical type, radiotherapy status, and PET/CT system across postoperative time intervals; Table S3: Characteristics of confirmed recurrent cases; Table S4: Patterns and sites of disease recurrence detected by FDG PET/CT; Table S5: Diagnostic performance according to the method of recurrence confirmation; Table S6: Diagnostic performance of the first postoperative FDG PET/CT scan for recurrent disease (patient-level analysis); Table S7: Comparison of FDG uptake between postsurgical changes and local recurrent lesions (patient-level analysis).

## Abbreviations

The following abbreviations are used in this manuscript:

BP	blood pool
CTx	chemotherapy
CT	computed tomography
EPP	extrapleural pneumonectomy
FDG	^18^F-fluorodeoxyglucose
IRB	Institutional Review Board
L	liver
lesion/BP ratio	local recurrent lesion SUVmax to blood pool SUVmean
lesion/L ratio	local recurrent lesion SUVmax to liver SUVmean
MPM	malignant pleural mesothelioma
NPV	negative predictive value
OP/BP ratio	postsurgical bed activity SUVmax to blood pool SUVmean
OP/L ratio	postsurgical bed activity SUVmax to liver SUVmean
P/D	pleurectomy/decortication
PET	positron emission tomography
PPV	positive predictive value
RTx	radiotherapy
SD	standard deviation
SUV	standardized uptake value
SUVmax	maximum standardized uptake value
SUVmean	mean standardized uptake value
VOI	volume of interest
