# Supplementary material for: FDG PET/CT for Postoperative Surveillance in Malignant Pleural Mesothelioma: Temporal Evolution of Postsurgical Metabolic Activity and Diagnostic Performance for Recurrence Detection
Source: Cancers (Basel). 2026 Jun 19;18(12):2000. doi: 10.3390/cancers18122000 (PMC13296393; doi:10.3390/cancers18122000)
Supplement: Supplementary file 1 [file cancers-18-02000-s001.zip › cancers-4369872-supplementary.pdf]

Supplementary Tables

Table S1. Postsurgical FDG uptake by time interval (patient-level analysis)

Time interval	N	OP/BP ratio	OP/L ratio
P1 (<6 months)	19	2.9 ± 1.6 (1.6–8.5)	2.1 ± 1.0 (1.0–5.6)
P2 (6–<12 months)	16	2.8 ± 1.5 (1.1–6.2)	1.9 ± 0.9 (0.8–4.0)
P3 (12– <24 months)	8	2.6 ± 1.5 (0.9–5.3)	1.8 ± 1.1 (0.7–3.8)
P4 (≥24 months)	1	2.3	1.9
P-value		0.903	0.669

Values are presented as mean ± standard deviation (SD). P4 included only one patient; therefore, statistical interpretation should be made with caution.

OP/BP ratio, postsurgical bed activity SUVmax to blood pool SUVmean; OP/L ratio, postsurgical bed activity SUVmax to liver SUVmean

Table S2. Distribution of histological subtypes, surgical type, and radiotherapy status, and PET/CT system across postoperative time intervals

	P1 (<6 months)	P2 (6–<12 months)	P3 (12–<24months)	P4 (≥24 months)	Total
Total scans	20	21	29	21	91
EPP	13 (65%)	15 (71.4%)	19 (65.5%)	17 (81%)	64 (70.3%)
P/D	7 (35%)	6 (28.6%)	10 (34.5%)	4 (19%)	27 (29.7%)
Epthelioid	13 (65%)	12 (57.1%)	19 (65.5%)	14 (66.7%)	58 (63.7%)
Biphasic	6 (30%)	8 (38.1%)	8 (27.6%)	6 (28.6%)	28 (30.8%)
Sarcomatoid	1 (5%)	0 (0%)	2 (6.9%)	1 (4.8%)	4 (4.4%)
Desmoplastic	0 (0%)	1 (4.8%)	0 (0%)	0 (0%)	1 (1.1%)
RTx Yes	12 (60%)	10 (47.6%)	16 (55.2%)	14 (66.7%)	52 (57.1%)
RTx No	8 (40%)	11 (52.4%)	13 (44.8%)	7 (33.3%)	39 (42.9%)
Biograph TruePoint	10	13	18	11	52
Discovery 710	10	8	11	10	39

EPP, extrapleural pneumonectomy; P/D, pleurectomy/decortication; RTx, radiotherapy

Table S3. Characteristics of confirmed recurrent cases (n = 49)

		Number of cases (%)
**Histologic subtype**	Epithelioid	25 (51.0)
	Biphasic	20 (40.8)

	Sarcomatoid	4 (8.2)
	Desmoplastic	0 (0)
**Surgical type**	EPP	36 (73.5)
	P/D	13 (26.5)
**Time interval**	P1 (<6 months)	7 (14.3)
	P2 (6–<12 months)	9 (18.4)
	P3 (12–<24 months)	17 (34.7)
	P4 (≥24 months)	16 (32.7)
**Treatment after recurrence defection**	Chemotherapy	22 (44.9)
	Radiotherapy	7 (14.3)
	Combined chemoradiotherapy	4 (8.2)
	Supportive care	16 (31.7)

EPP, extrapleural pneumonectomy; P/D, pleurectomy/decortication

Table S4. Patterns and sites of disease recurrence detected by FDG PET/CT (n = 49)

	**Number of recurrent lesions detected on PET/CT (%)**
**Recurrence pattern**	
Local only	18 (36.7)
Local + regional lymph node	8 (16.3)
Local + distant	8 (16.3)
Local + regional lymph node + distant	10 (20.4)
Regional lymph node + distant	2 (4.1)
Distant only	3 (6.1)
**Recurrence site**	
Local recurrence	44 (89.8)
Lymph node	
Intrathoracic*	19 (38.8)
Supraclavicular	6 (12.2)
Distant	
Intrathoracic metastasis	
Lung nodule	6 (12.2)
Contralateral hemithorax	2 (4.1)
Extrathoracic metastasis	
Abdominal lymph node	4 (8.2)
Peritoneum	9 (18.4)

Muscle	4 (8.2)
Bone	3 (6.1)
Liver	1 (2.0)
Colon	1 (2.0)
Oral cavity	1 (2.0)

* Intrathoracic includes bronchopulmonary, hilar, internal mammary, peridiaphragmatic, pericardial fat pad, or intercostal lymph nodes.

FDG PET/CT, ^{18}F-fluorodeoxyglucose positron emission tomography/ computed tomography

Table S5. Diagnostic performance according to the method of recurrence confirmation

	Total scan (n = 91)	Histopathological confirmation (n =11)	Radiological follow-up (n =80)
True positive	49	8	41
True negative	40	1	39
False positive	2	2	0
False negative	0	0	0
Sensitivity	100%	100%	100%
Specificity	95.2%	33.3%	100%
Positive predictive value	96.1%	80.0%	100%
Negative predictive value	100%	100%	100%
Accuracy	97.8%	81.8%	100%

Table S6. Diagnostic performance of the first postoperative FDG PET/CT scan for recurrent disease (patient-level analysis)

**Diagnostic performance**	**Value**
True positive	19
True negative	25
False positive	1
False negative	0
Sensitivity	100.0% (19/19)
Specificity	96.2% (25/26)
Positive predictive value	95.0% (19/20)
Negative predictive value	100.0% (25/25)
Accuracy	97.8% (44/45)

Table S7. Comparison of FDG uptake between postsurgical changes and local recurrent lesions (patient-level analysis)

**Parameters**	**Postsurgical uptake**	**Local recurrent lesions**	**P-value**
Blood pool (BP) ratio	2.8 ± 1.5 (0.9–6.4)	4.1 ± 1.8 (1.6–7.9)	0.004
Liver (L) ratio	2.0 ± 0.9 (0.6–4.1)	2.8 ± 1.3 (1.1–5.8)	0.006

Patient-level analysis was performed using the first postoperative PET/CT scan per patient. Values are presented as mean ± standard deviation (SD).

BP ratio, SUVmax normalized to mediastinal blood pool SUVmean; L ratio, SUVmax normalized to liver SUVmean
